# Evaluation of the structural changes of uveitis patients by optical biometry

**DOI:** 10.22336/rjo.2023.40

**Published:** 2023

**Authors:** Ahmet Kürşad Sakallioğlu, Goksu Alacamli, Samira Sattarpanah, Hande Guclu

**Affiliations:** * Department of Ophthalmology, Clinical County Emergency Hospital, Brăila, Romania; ** Department of Ophthalmology, “Grigore T. Popa” University of Medicine and Pharmacy, Iaşi, Romania

**Keywords:** anterior segment, optical biometry, uveitis

## Abstract

**Objective (Aim):** To observe the ocular structural changes in active and inactive uveitis patients.

**Methods:** This retrospective study involved 30 patients (32 eyes) with anterior and intermediate uveitis cases and 54 eyes of 54 cases in a control group, who were admitted to the Ophthalmology Department at Trakya University. In the study group, 14 patients were females, 16 patients were males and in the control group 26 volunteers were females, and 28 volunteers were male of the 54 volunteers. Anterior chamber depth, axial length, intraocular pressure, lens thickness, central corneal thickness, steep and flat values in keratometry, corrected visual acuity in both eyes, anterior chamber cells, and vitreous cells were measured and compared between three groups (two uveitis groups - active and inactive - and control group).

**Results:** In the comparison of the median values of axial length, central corneal thickness, and steep and flat values of keratometry, the values of the patients with active uveitis were higher than the ones in the control group in each parameter, but no significant difference was observed. The anterior chamber depth parameter value was higher, the lens thickness value was lower in patients with active uveitis than the values in the control group and the differences were statistically significant (p<0,05). No significant structural differences in the values of the active and inactive group patients (p>0,05) were observed.

**Conclusions:** Only lens thickness and anterior chamber depth parameters were statistically significant in patients with active uveitis, compared with the inactive uveitis group. Anterior chamber depth measurement values were higher and lens thickness measurement values were lower in patients with active uveitis when compared with the control group.

**Abbreviations: **AAU = Acute anterior uveitis, CAU = Chronic Anterior Uveitis, AC = Anterior Chamber, IOP = Intraocular Pressure, IVCM = *in vivo* Confocal Microscopy, AS-OCT = Anterior Segment Optical Coherence Tomography, UBM = Ultrasound Biomicroscopy, LFP = Laser Flare Photometry, KP = Keratic Precipitates, OCT = Optical Coherence Tomography, AL = Axial Length, ACD = Anterior Chamber Depth, LT = Lens Thickness, CCT = Central Corneal Thickness, Ks = Steep Value of Keratometry, Kf = Flat Value of Keratometry, AUP = Active Uveitis Patients, IUP = Inactive Uveitis Patients, SUN = Standardization of Uveitis Nomenclature

## Introduction

Uveitis is a diverse group of inflammatory diseases with varying frequencies worldwide based on geography [**1**].

Anterior uveitis is the most common type of uveitis [**2**-**4**]. Acute anterior uveitis (AAU) occurs more frequently than chronic anterior uveitis (CAU) or posterior uveitis [**5**]. Acute uveitis typically presents with pain, redness in the eye, and inflammation in the anterior chamber (AC). It may also cause increased intraocular pressure (IOP) [**6**]. Uveal inflammation can affect any tissue or cavity within the eye. Some effects may be temporary and not respond to medical treatment, while others may be permanent [**7**]. Edema in the cornea is a possible result of inflammation or high IOP. Inflammatory debris, posterior synechiae, cataract formation on the lens, cells, and flare in AC, exudate, and hypopyon in humor aqueous, posterior synechiae, pupil distortion, atrophy on iris are some possible effects of uveitis [**1**].

A variety of ophthalmic imaging techniques have been available for anterior segment analysis for a long time, such as anterior segment photography, specular microscopy, *in vivo* confocal microscopy (IVCM), anterior segment optical coherence tomography (AS-OCT), ultrasound biomicroscopy (UBM), laser flare photometry (LFP), and less commonly used techniques, such as anterior segment angiography, iris autofluorescence, and infrared imaging. Imaging techniques enhance the evaluation of structural changes in anterior uveitis. In recent studies, multiple methods have been used to analyze structural changes in eyes [**8**]. In 2004, Wertheim et al. [**9**] published a report on the architecture of keratic precipitates (KP) in patients with ocular inflammation caused by infectious and noninfectious factors. Investigations continued IVCM outcomes of kP in different patients and uveal diseases [**10**]. In their study, Li Y et al. [**11**] have concluded that optical coherence tomography (OCT) provides more objective information about inflammatory cells in AC. The counts of OCT cells showed a strong correlation with the cell grades observed through the slit-lamp microscope. Optical coherence tomography can detect particles in the inferior region of the anterior chamber that clinical slit-lamp examination may miss. It could be a useful tool in managing anterior uveitis. Da Costa et al. [**12**] studied the correlation between the length of ciliary processes, measured through ultrasound biomicroscopy, and the severity, duration, and location of uveitis. As a result, they observed that ultrasound biomicroscopy is useful in evaluating anatomical changes of ciliary processes in uveitis and ocular hypotony, providing a basis for recommending baseline screening of uveitis patients. Agrawal et al. [**13**] classicized semi-automated ﬂare readings using the Kowa FM 700 laser cell ﬂare meter in patients with uveitis. The study proposed a classification system that quantifies the severity of flares, reducing ambiguity in the communication among clinicians and researchers. 

However, there are limited studies of optic biometry in uveitis patients. Therefore, in this study, the aim was to analyze the structural changes of the eye by the optical biometry measurements [anterior chamber depth (ACD), axial length (AL), lens thickness (LT), central corneal thickness (CCT), steep value of keratometry (Ks), and flat value of keratometry (Kf)] in active uveitis patients (AUP) and inactive uveitis patients (IUP), because optical biometry evaluation results might be useful to understand the anatomical changes during uveitis.

## Methods

 This retrospective comparative study analyzed 32 eyes of 30 patients with anterior and intermediate uveitis in the ophthalmology department of a university hospital, from June 2019 to June 2020. The control group included 54 eyes of 54 age and sex-matched healthy individuals who were admitted to the ophthalmology department for a routine evaluation. All patients in the study group were selected from those who had a uveitis attack for the first time between the specified dates. Patients who had other ocular comorbidities (such as diabetic retinopathy), a history of ocular trauma and ocular surgery, and patients who had other ocular inflammatory and structural disorders were excluded from the study.

The study followed the principles of the Declaration of Helsinki. Patients gave informed consent after receiving an explanation of the study’s nature and potential consequences. The Ethics Committee at the university hospital approved the study.

The criteria defined by the Standardization of Uveitis Nomenclature (SUN) Working Group were used to select uveitis patients and for anatomic classification, descriptors, grading AC cells and flare, activity terminology of uveitis of the patients [**14**]. The diagnosis of the disease was made by analyzing the patient’s medical history and the clinical findings of their ophthalmological examination.

All patients underwent a complete ophthalmological examination including corrected visual acuity with Snellen chart, biomicroscopic, IOP measurement with Goldmann applanation tonometer, and detailed fundus examination with a 78-diopter non-contact lens (Volk, Ohio, USA) at each visit. Swept-source optic coherence-based optical biometry (IOL Master 700, Carl Zeiss Meditec AG, Jena, Germany) was used to evaluate the anterior segment during the active uveitis attack in which the patients were diagnosed, and during the inactive uveitis period in which anterior segment cells were disappeared. The procedure was carried out without dilating the pupils in either case. AL, ACD, LT, CCT, and Kf-Ks were evaluated from the optical biometry measurements.

Clinical data were analyzed. The median value of each parameter was compared. The student t-test was used to compare the values of AUP and IUP with the control group at AL, CCT, and Kf-Ks parameters, and AUP with the control group at the ACD parameter. Mann-Whitney U test was used to compare values of AUP and IUP with the control group at LT, Ks, and IOP parameters, and the mean value of IUP with the control group at ACD parameter. Also, the Mann-Whitney U test was used to compare AUP values with IUP values at ACD, and LT parameters. Chi-square tests and the Independent Samples Test were used to compare the demographic features between the groups. Also, the Independent Samples Test was used to compare AUP values with IUP values at AL, CCT, Kf, Ks, and IOP parameters. P values < 0.05 were considered statistically significant. The statistical analysis was performed using commercially available software (SPSS, Version 18.0; SPSS Inc., Chicago, IL).

## Results

A total of 86 eyes belonging to 84 patients were examined and analyzed. In the study group of 30 patients, 14 (46.7%) were females and 16 (53.3%) were males. In the control group, of the 54 patients, 25 (46.2%) were females, 29 (53.8%) were males. The mean age of the study and the control group was 43.2 ± 17.9 (range 14-80), and 43.9 ± 19.5 (range 15-72) respectively. No significant difference between the two groups in terms of gender (p>0.05) and age (p>0.05) was observed. 

In the comparison of median AL, CCT, Kf values, and mean Ks value of the AUP with the control group, values of AUP were higher than the control group in each parameter, but no significant difference (p>0.05) was observed. Also, the mean IOP values were lower in the AUP group than the mean IOP values of the control group and this difference was not statistically significant. However, the median ACD value was significantly higher, and the mean LT value was significantly lower in the AUP group compared to the values of the control group (**Table 1**). No significant structural differences between AUP and IUP (p>0,05) were observed. 

**Table 1:** Statistical analysis of the biometric values of the patients with active uveitis values and the values of the control group

Parameter	AUP mean/median values	Control group mean/median values	P-value
AL	23,59 ± 1,07	23,34 ± 0,83	0,242
ACD	3,62 ± 0,53	3,18 ± 0,5	< 0,001*
LT	3,88 (3,42-4,30)	4,65 (4,36-4,87)	< 0,001*
CCT	548 ± 31,87	539,89 ± 36,63	0,313
Kf	43,15 ± 1,32	42,82 ± 1,31	0,276
Ks	44,21 (43,53-45,14)	43,87 (42,85-44,91)	0,267
IOP	14 (12-16)	15 (12-16)	0,411
AUP = active uveitis patients; AL = axial length; ACD = anterior chamber depth; LT = lens thickness; CCT = central corneal thickness; Kf = keratometry flat; Ks = keratometry steep; IOP = intraocular pressure			
*Statistically significant value			

Median AL, CCT, Kf, values and mean Ks value were higher in IUP than the control group in each parameter, but statically, no significant difference was observed. In addition, the mean IOP values were lower in the IOP group than in the control group, but this difference was not significant. Also, the mean IOP value was lower in the IUP group than in the control group, but this difference was not significant. However, the mean ACD value was significantly higher, and the mean LT value was significantly lower in the IUP group compared to the control group (**Table 2**). 

**Table 2:** Statistical analysis of the biometric values of the patients with inactive uveitis values and the values of the control group

Parameter	IUP mean/median values	Control group mean/median values	P-value
AL	23,59 ± 1,02	23,34 ± 0,83	0,246
ACD	3,58 (3,32-3,78)	3,13 (2,78-3,34)	< 0,001*
LT	3,95 (3,48-4,38)	4,65 (4,36-4,87)	< 0,001*
CCT	542,7 ± 34,93	539,89 ± 36,63	0,733
Kf	43,16 ± 1,33	42,82 ± 1,31	0,268
Ks	44,19 (43,75-45,06)	43,87 (42,85-44,91)	0,188
IOP	14 (12-15,75)	15 (12-16)	0,077
IUP = inactive uveitis patients; AL = axial length; ACD = anterior chamber depth; LT = lens thickness; CCT = central corneal thickness; Kf = keratometry flat; Ks = keratometry steep; IOP = intraocular pressure			
*Statistically significant value			

**Fig. 1:**
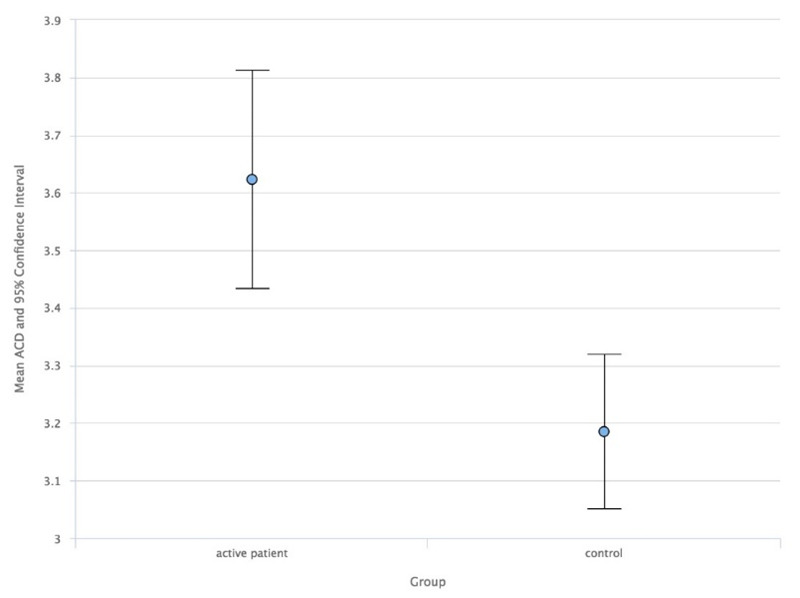
Median values of ACD in AUP and control group

**Fig. 2:**
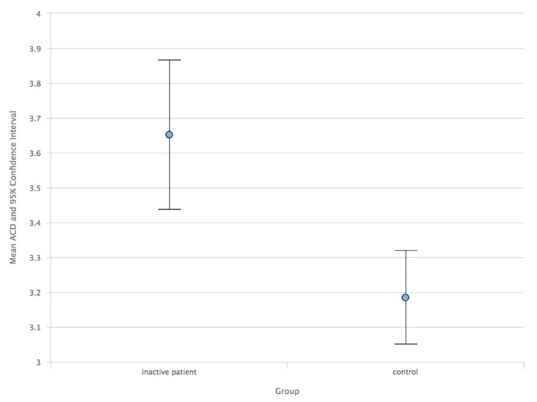
Median values of ACD in IUP and control group

**Fig. 3:**
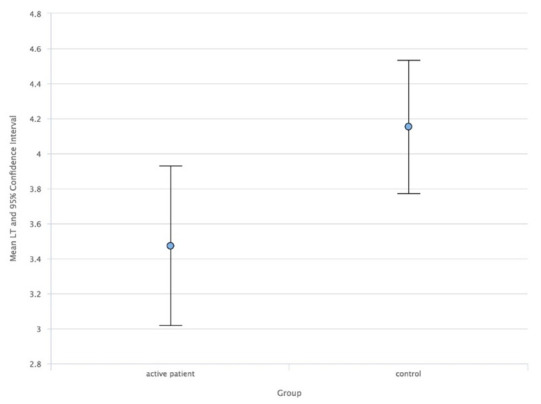
Mean values of LT in AUP and control group

**Fig. 4:**
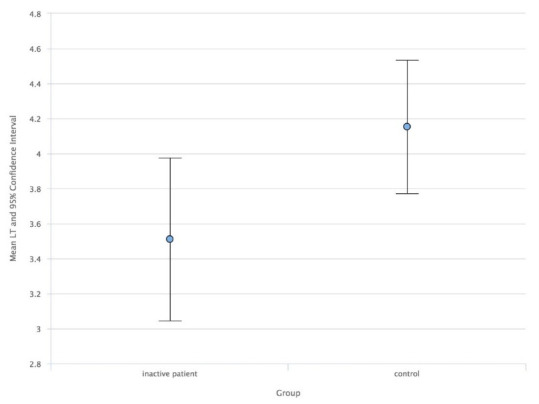
Mean values of LT in IUP and control group

As a result, comparing the study group with the control group, the ACD median values in AUP (**Fig. 1**) and ACD mean values in IUP (**Fig. 2**) were higher than in the control group. Also, the LT mean values in AUP (**Fig. 3**) and IUP (**Fig. 4**) were lower than in the control group. Only these comparisons were statistically significant. Also, no significant structural differences were determined between AUP and IUP.

## Discussion

In this study, a significant difference in LT and ACD measurements was observed between the study and the control groups compared with optical biometry. In the study group, AC was deeper and the lens was thinner than the values of the control group in both AUP and IUP. Based on the intraocular changes well known during uveitis, we developed our hypothesis regarding this result as follows.

In trabeculitis, inflammation and steroid use can increase eye pressure due to reduced drainage and adhesions [**15**]. Also, increased cells and protein exudates in humor aqueous cause vision loss [**1**].

Mechanical blockage of the trabeculae due to serum components liberated by vascular incompetence is the proposed mechanism [**16**]; prostaglandin-mediated vascular hyperpermeability results in hypersecretion [**17**,**18**]; the protein that interferes with active transport overtaxes the outflow mechanisms [**19**]; inflammation of the trabeculae itself with swelling that causes impaired outflow [**20**]; an inflammatory process that damages the trabecular endothelial cells [**21**]; precipitates on the meshwork that obstruct outflow mechanically [**22**]; sclerosis of trabecular meshwork results in a chronic inflammation [**23**] or hyaline membrane obstructs the trabeculae [**23**]. Due to the influence of these mechanisms, the effects of uveitis on the humor aqueous outflow reduction was one of the reasons for the deep AC, while IOP measurements were within normal limits. The other hypothesis refers to hypersecretion associated with prostaglandin-mediated vascular hyperpermeability, which leads to an inflow of extra humor aqueous and also proteins and inflammatory cells. These components cause increased ACD. 

The lens capsule and epithelium regulate the flow of aqueous humor to the lens fibers and act as a regulatory barrier [**24**]. This allows for the passive exchange of metabolic substrates, waste expulsion, and selective molecule filtering based on size and charge [**24**-**28**]. The uveitic capsules show extensive and varied structural changes due to ocular inflammation and epithelial-mesenchymal transition [**29**]. It is believed that these changes in the structure of the lens capsule and epithelium make it easier for water, ions, and possibly larger molecules to pass through the basement membrane [**28**-**30**]. In our study, LT was measured lower than the control group. We hypothesized that this facilitation leads to an increased outflow of the inner lens substances to humor aqueous. Therefore, these reasons may explain our decreased measurements of LT. 

Szepessy et al. [**31**] investigated anterior segment characteristics of Fuchs uveitis syndrome with a Scheimpflug imaging camera and they found that the central cornea was thinner, the iridocorneal angle was significantly larger, and the ACD was deeper in the eyes with Fuchs Uveitis Syndrome. In our study, CT changes were not significant, but the ACD was deeper. 

Bayrak et al. [**32**] evaluated crystalline lens density using Scheimpflug lens densitometry in different uveitis entities and they concluded that at 3 months, Pentacam densitometry of zones 3 values was determined to be significantly high in the uveitic eyes. The average lens density value at 1 month was statistically significantly higher than the baseline value in the uveitic eyes. In our study, LT was thinner than the control group. Not surprisingly, a reduction in LT and volume increases lens density. The results of our study are in line with this study.

A significant reduction in central and pericentral corneal thickness was observed by Agra et al. [**33**], but patients did not exhibit a significant change in IOP during the disease on AS-OCT fifteen days after the treatment of eyes with acute AAU. In our study, no changes were observed in CCT. However, as in this study, the changes in IOP were not significant, supporting our study.

## Conclusion

The present study aimed to observe the structural changes of the human eye in uveitis. A small sample size was studied in the study and it represented the limitation. Certainly, further studies including a larger number of patients would explain the structural changes of the human eye and the mechanism better. We believe that studies on this subject have not been conducted enough until the present. We determined remarkable changes in ACD and LT in the anterior segment. The scarcity of optical biometry measurements on uveitis patients in literature makes the study valuable. Certainly, further studies that will explain the structural changes of the human eye and the mechanism are required.

**Conflict of Interest**

The authors state that they do not have any conflicts of interest.

**Informed Consent and Human and Animal Rights statements**

Written informed consent has been obtained from the individuals involved in the study.

**Authorization for the use of human subjects**

Ethical approval: The research related to human use complies with all the relevant national regulations and institutional policies, is in accordance with the tenets of the Helsinki Declaration, and has been approved by the research ethics committee of Trakya University, Faculty of Medicine, Turkey (approval code: TÜTF-BAEK 2020/400).

**Acknowledgments**

None.

**Sources of Funding**

The author(s) received no financial support for the research, authorship, and/or publication of this article.

**Disclosures**

None.
